# A neuroeconomic signature of opioid craving: How fluctuations in craving bias drug-related and nondrug-related value

**DOI:** 10.1038/s41386-021-01248-3

**Published:** 2021-12-16

**Authors:** Kathryn Biernacki, Silvia Lopez-Guzman, John C. Messinger, Nidhi V. Banavar, John Rotrosen, Paul W. Glimcher, Anna B. Konova

**Affiliations:** 1 Department of Psychiatry, University Behavioral Health Care, and the Brain Health Institute, Rutgers University–New Brunswick, Piscataway, NJ 08855 USA; 2 Neuroscience Institute, NYU Grossman School of Medicine, New York, NY 10016 USA; 3 Grupo de Investigación en Neurociencias (NeURos), Escuela de Medicina y Ciencias de la Salud, Universidad del Rosario, 111221 Bogotá, Colombia; 4 Harvard Medical School, Boston, MA 02115 USA; 5 Department of Cognitive Sciences, University of California–Irvine, Irvine, CA 92697 USA; 6 Department of Psychiatry, NYU Grossman School of Medicine, New York, NY 10016 USA

**Keywords:** Decision, Addiction

## Abstract

How does craving bias decisions to pursue drugs over other valuable, and healthier, alternatives in addiction? To address this question, we measured the in-the-moment economic decisions of people with opioid use disorder as they experienced craving, shortly after receiving their scheduled opioid maintenance medication and ~24 h later. We found that higher cravers had higher drug-related valuation, and that moments of higher craving within-person also led to higher drug-related valuation. When experiencing increased opioid craving, participants were willing to pay more for personalized consumer items and foods more closely related to their drug use, but not for alternative “nondrug-related” but equally desirable options. This selective increase in value with craving was greater when the drug-related options were offered in higher quantities and was separable from the effects of other fluctuating psychological states like negative mood. These findings suggest that craving narrows and focuses economic motivation toward the object of craving by selectively and multiplicatively amplifying perceived value along a “drug relatedness” dimension.

## Introduction

The opioid epidemic is a health crisis with disastrous personal and societal costs. Reuse and relapse in opioid use disorder (OUD) is driven in part by craving—the fluctuating, intense, and specific desire for the drug. Craving is an extensively studied and well-established predictor of drug use [1–20]. Yet, we still lack a precise understanding of how the psychological experience of craving translates to pursuit of the drug over other valuable, and healthier, alternatives. Unfortunately, gold-standard OUD treatments (e.g., methadone) do not fully eliminate craving and fail to prevent reuse in many patients [4, 19, 21]. Identifying, and ultimately mitigating, the mechanistic source of craving offers one avenue to monitor and treat OUD more effectively.

We recently developed an experimental approach to quantify how specific craving (e.g., for a chocolate bar) is reflected in decision-making processes [22]. We found that, compared to a baseline of no craving, craving for a specific snack food caused people to pay more for the craved food and for subjectively similar foods, but not for dissimilar options. This shift in subjective valuation was short-lived with a measurable half-life of <1 h. Further, it was well-described by a multiplicative-gain process: craving and amount of the food available were integrated in a nonlinear fashion to “tune” a food’s subjective value. When the craved (and similar) foods were available in larger quantities, willingness-to-pay increased even more, indicating craving *selectively* and *disproportionately* increased these options’ values. This suggested (1) craving operates along an attribute-similarity dimension, based on proximity to the object of craving; and that it (2) interacts with valuation (vs. being a separable signal that is “added” to baseline value), by scaling a person’s internal value representation—their utility function in economic terms—for these shared attributes. These characteristics provide an algorithmically precise signature of craving that can explain how, by acting on normal valuation mechanisms, craving modifies specific value and selectively biases decisions toward the object of craving.

**Fig. 1:**
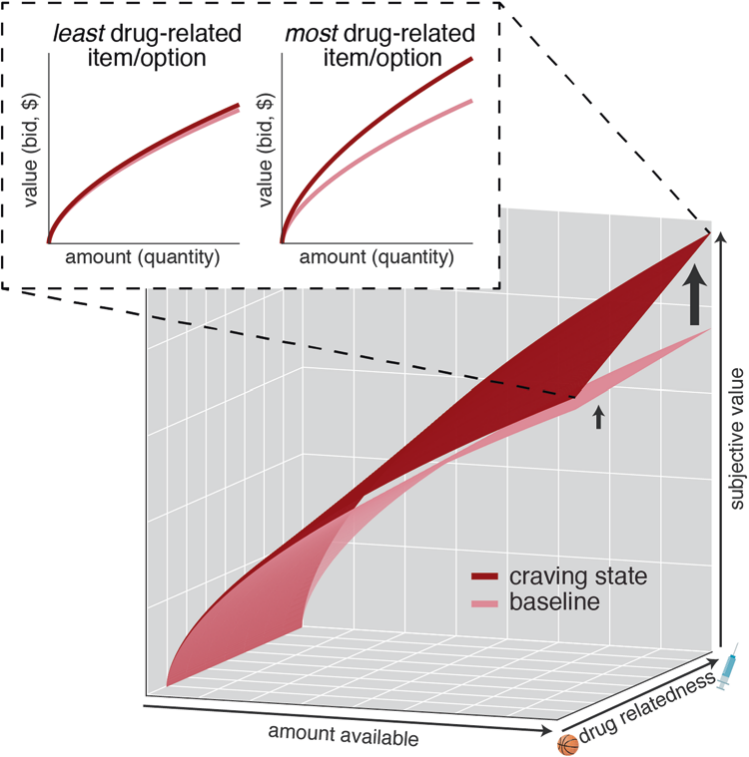
Hypothesized model of drug craving. Drug craving in opioid use disorder, like food craving in healthy people, is hypothesized to act on normative valuation mechanisms: opioid craving should selectively amplify the value of options judged as more drug related, and more so when these options are available in higher quantities. This interaction between drug-relatedness, amount, and craving may explain how acute psychological craving biases decisions toward drug seeking and use.

Here, we tested whether the same selective and multiplicative process underlies drug craving, specifically craving for opioids in treatment-engaged OUD patients for whom craving represents a clinically significant barrier to recovery. Given that drug craving is closely tied to cue reactivity, we reasoned that a key attribute dimension in drug craving may be drug associability or relatedness, such that options judged as more related to the drug are imbued with higher value during craving (Fig. 1).

Prior work indicates acute craving for alcohol and nicotine markedly increases behavioral economic demand indices of drug value [23]: people experiencing craving are willing to purchase the craved drug—in a real or hypothetical sense—at higher costs and to consume more of it when it is “free” [24–29]. Similar changes in drug value have been observed following 24 h abstinence from opioid medications in OUD [30, 31]. However, these studies have not typically tested whether craving *selectively* affects drug value nor have they provided a compact model of the underlying process. The few previous studies that have examined the acute impact of craving on alternative nondrug-related outcomes yielded conflicting findings [31–35], and are limited by the use of monetary reward for this purpose (which is associated with drug-seeking and use). The hypothetical nature of many of these studies may also limit inference based on individuals’ “true” preferences as craving evolves.

Craving for drugs, particularly opioids, co-occurs with a mixture of (mostly negative) affective experiences [36–38], sometimes referred to as ‘hyperkatifeia’ [39–41]. However, negative mood has incidental effects in decision-making [42–44], and could be expected to shift value globally rather than specifically for the drug, increasing the attractiveness of both drug- and nondrug-related options. To isolate the specific effect of craving in valuation and advance a mechanistic understanding of how craving biases decisions toward drug-seeking actions in OUD, it may also be necessary to separate craving from negative mood.

In a within-subjects crossover design, here, OUD patients completed a hybrid symptom-capture/willingness-to-pay paradigm, both after receiving their scheduled methadone and ~24 h after the last dose of methadone, a manipulation found previously to promote drug cue reactivity and craving [45]. Willingness-to-pay is related to traditional behavioral economic demand indices but has the key advantage that it can be used to estimate values in a single trial, without the need to summarize data across dozens of trials during which craving levels may be changing. We used this hybrid task to (1) repeatedly sample a person’s “true” (consequential) value for (2) a range of choice options spanning a personalized drug relatedness dimension, orthogonal to key features such as general desirability, while (3) simultaneously and repeatedly assessing their psychological state of craving and other transitory states.

## Materials and methods

### Participants

Participants were 35 individuals with DSM5-defined OUD, as determined by clinic staff and obtained from patient charts, for whom heroin use was primary and heroin craving was persistent in the past 12 months (i.e., all endorsed the DSM5 “Craving” criterion). All participants were ≥18 years of age, fluent English speakers, and enrolled in an outpatient methadone treatment program, from which they were directly recruited. Informed consent was obtained in accordance with procedures approved by the NYU School of Medicine IRB.

Exclusion criteria were: (1) primary drug of choice other than opioids; (2) head trauma, loss of consciousness >30 min, or neurologic disease; (3) unstable or untreated medical conditions (e.g., late-stage HIV/AIDS); and (4) unstable or untreated psychiatric conditions such as a current manic episode, active psychosis, or suicidal ideation. Inclusion/exclusion criteria and sample characterization were ascertained during a comprehensive interview that included the Addiction Severity Index [46], DSM5 substance use disorders checklist, and Beck Depression [47], and Anxiety [48] Inventories, all administered by the experimenters, and by consulting patient charts. Of the 35 participants consented, 6 were excluded due to problems confirming methadone dosing on testing days/randomization failure, leaving *N* = 29 included for analysis (age = 47.38 [*SD* = 11.53] years, 89.7% male, receiving a mean methadone dose = 92.24 [*SD* = 31.33] mg; Table S1).

### Study design

**Fig. 2:**
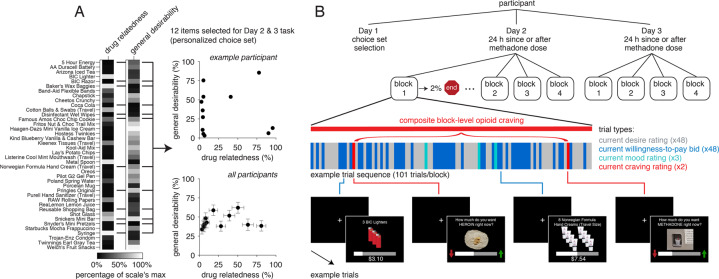
Experimental design. **A** On *Day 1*, participants rated each of 40 common consumer items and snack foods on their subjective drug relatedness and general desirability, among other dimensions. For each participant, these ratings were used to sub-select a 12-item choice set that (1) provided maximal spread in drug relatedness (that was of main interest here), while (2) de-coupling this dimension from general desirability thus minimizing the possibility of a systematic bias in the desirability of the items chosen as drug-related or nondrug-related (see Supplementary Information for details on selection of the initial 40-item fixed set and the choice set personalization procedure). The final selected choice sets only partly overlapped across participants, revealing a high degree of subjectivity in drug relatedness judgements (Fig. S1). **B** Following screening and choice set selection on *Day 1*, participants completed two task sessions, one after taking their daily methadone dose and the other ~24 h since the last dose of methadone (*Days 2* and *3*, randomized crossover order). Each task session was composed of 4 blocks. Each block was composed of 101 trials. Trials were randomly presented and consisted of 48 willingness-to-pay bid trials for each of the 12 items in the personalized choice set presented in 4 different quantities (depicted in blue), 48 desire rating trials for each of the same 12 items × 4 quantities (gray), 3 mood rating trials for each of happiness, stress, and boredom (teal), and 2 craving rating trials for each of heroin and methadone (red). For purposes of analysis, the three mood state ratings were averaged for each block into a composite negative mood measure and the two craving ratings were averaged into a composite opioid craving measure. To incentivize participants to provide their “true” momentary value (i.e., bid) for each item and to report only on their current subjective experience in each block, the task was structured such that it could end with a fixed (2%) probability after each block in a session, ending regardless after the 4^th^ block. At the end of the session, one bid trial from the last completed block was randomly selected and played out to determine a participant’s bonus.

Each participant completed up to three study sessions (Fig. 2). In the first session (*Day 1*), participants completed clinical assessments, a ratings task that probed multiple dimensions for each of 40 items to be used in the willingness-to-pay task completed at each of two additional sessions, and instructions and practice for the task. In the second and third sessions (*Days 2* and *3*), participants completed the willingness-to-pay task, followed by two other decision-making tasks (not discussed here). Participants received $20 for the first session and $30 for each of the two task sessions, plus a bonus determined by a single, randomly selected choice made during that session (see below and Supplementary Information). To introduce variability in opioid craving [30, 45], participants were randomized to complete one of the two task sessions after taking their scheduled daily methadone (mean = 1.63 [*SD* = 1.44] h), and the other ~24 h after the last dose of methadone (mean = 24.84 [*SD* = 9.28] h), in the window of peak and trough of methadone levels, respectively. Compliance with the treatment manipulation was confirmed via self-report and time-stamped medication dispensing records obtained from the methadone program. The two task sessions were separated by 12.2 (*SD* = 18.76) days. All *N* = 29 participants completed the first session and at least one of the two task sessions; 23 completed all three sessions, for a total of 81 sessions included for analysis. Of the 6 participants that completed only one task session, 3 did so 24 h after methadone dosing, and 3 shortly after methadone dosing (randomization preserved). There was no systematic dropout related to the study procedures. Reasons for non-completion included treatment dropout, hospitalization, and/or scheduling conflicts. Apart from a slight increase in withdrawal 24 h after methadone dosing, there were no baseline differences in clinical status across the three study days (Table S2).

### Choice set selection (Day 1)

Because cues associated with drug use and craving are highly subjective [49, 50], we developed personalized choice sets for each participant, selected from a broader fixed set. Participants rated 40 consumer items and snack foods on five subjective dimensions: drug relatedness, (general) desirability, frequency of use/consumption, tastiness of snacks, and healthiness of snacks. Ratings used a continuous rating scale, which varied based on the dimension probed (e.g., “Not at all likely [to make me think of using heroin and other drugs]” to “Extremely likely [to make me think of using heroin and other drugs]” or “Not at all [desirable]” to “Extremely [desirable]”). Responses from the ratings task were used to sub-select 12 consumer items and snack foods (personalized for each individual) to be offered during the willingness-to-pay task completed in subsequent sessions. Choice sets were selected to ensure maximal spread in the drug relatedness dimension while maintaining little or no correlation between general desirability and drug relatedness, by iteratively and randomly selecting 12-item sets (6 consumer items, 6 snack foods) and computing the correlation between the two ratings until a sub-set met a *P* > 0.25 cut-off (equivalent to *R* < | 0.2058 | , or small-to-moderate association). This procedure aimed to minimize potential confounding effects due to differences in the general value/desirability of the more/less drug-related items, as confirmed in post-hoc analyses showing general desirability did not significantly relate to drug relatedness for the final 12-item choice set selected for each participant (*B* = 0.06, *t*_105.15_ = 1.12, *P* = 0.27). Supplementary Information provides detailed instructions given to participants for the ratings tasks and additional details on the choice set optimization procedure, and Fig. 2A shows an illustrative example of the latter.

### Willingness-to-pay task (Days 2 and 3)

Participants completed a modified task [22] composed of bid trials (our measure of subjective value), desire rating trials, and mood/craving rating trials. In *bid trials*, participants indicated their willingness to pay in the current moment (from $0 to $15 in $0.02 increments) for each item in their 12-item choice set, offered one at a time in one of four quantities (1, 3, 5, or 8 units). For these trials, participants were explicitly instructed to bid the maximum amount they would pay right now for the item(s) shown, for the real chance of getting the item(s) right now. In *desire rating trials*, participants indicated their desire in the current moment for each item, again offered in different quantities. Specifically, they were instructed to indicate how much they want the item(s) shown right now, without any consequence for whether or not they can have the item(s) right now (non-contingent response). As in our prior study, desire ratings were included to standardize the strategy/cognitive process participants engaged in placing their bids (i.e., they were encouraged to base their bids on their current desire for each item rather than e.g., market prices). In *mood rating trials*, participants reported on their current feelings of boredom, stress, and happiness. In *craving rating trials*, current desire for heroin and methadone was used to evaluate overall opioid craving. All rating trials employed a continuous rating scale presented below a picture cue identifying the rating type (see Fig. 2B and Supplementary Information for detailed instructions given to participants for each trial type).

In each task block (maximum of 4 per session), participants submitted one bid and one desire rating for each item and quantity (*N* = 2 × 12 ×4 = 96), and one rating for each mood/craving (*N* = 3 + 2 = 5), for 101 trials/block. Trials were self-paced, separated by a variable 0.5–1.0 s inter-trial-interval, and fully randomized within a block (Fig. 2B). To capture participants’ “true” momentary valuation in an incentive-compatible manner, we implemented a fixed hazard rate such that the task had 2% chance of ending after each block, ending after the 4^th^ block regardless. At the end of each session/day, one bid trial was randomly selected from the last completed block and realized using an auction procedure to determine a participant’s bonus, in the form of money, the item(s) shown on that trial, or both. Both the (small) probability of the task ending early and the auction procedure were fully described to participants (see Supplementary Information). In total, participants completed an average of 710 trials (~7 blocks) across the two task sessions/days.

### Data analysis

Our primary analytic approach was linear mixed effects regression, with bid for item *i*, participant *j*, and trial *t* as the outcome variable. Because momentary (block-to-block) methadone craving strongly positively correlated with heroin craving (*P* = 3.9 × 10^–15^), and to increase robustness of our block-level estimate of craving given only a single randomly interspersed craving rating per drug type was collected in each block, the two ratings were averaged into a composite “opioid craving” measure for analysis. Similarly, block-to-block reported stress, the inverse of happiness, and boredom were correlated (*P* = 0.04–5.7 × 10^–7^) and combined into a composite “negative mood” measure. The initial model assessed whether participants’ bids were predicted by an item’s drug relatedness, participant-mean level of reported opioid craving, participant mean-centered craving in the local task block, and the interaction between these factors (*Model 1*). This permitted separately assessing between-person and within-person contributions of craving to drug-related valuation, respectively [51]. Potential effect modifiers were also explored building on this model, and included study day, item general desirability, and between- and within-person negative mood. To further probe the mechanism of anticipated value change, item quantity and its interactions with drug relatedness and craving were added as additional predictors (*Model 2*; Table 1). Models were estimated in MATLAB (*fitlme*) and R (*lme4*) and included random intercepts and random slopes for drug relatedness (which differed across participants but remained constant across days/blocks; *Models 1* and *2*) and quantity (*Model 2*) by participant. Degrees of freedom for significance testing were computed using Satterthwaite approximation.

**Table 1:** Association of patients’ level of current reported opioid craving, item drug relatedness, and item quantity with bids ^a^.

**Model 1a: Effect of craving and item drug relatedness on bids**						
AIC	46359.5					
BIC	46431.4					
Num. of observations	9776					
		***B***^b^	***SE***	***t*–stat**	***df*** ^c^	***P*–value**
(Intercept)		−3.06	0.69	4.43	28.69	0.0001
Drug relatedness		−1.95	0.46	−4.19	25.45	0.0003
Opioid craving (person-mean)		1.26	1.62	0.78	28.69	0.44
Opioid craving (person-centered)		0.71	0.18	3.90	9720.47	9.57 × 10^–5^
Drug relatedness × opioid craving (person-mean)		2.56	1.12	2.29	26.71	0.03
Drug relatedness × opioid craving (person-centered)		1.21	0.40	2.99	9720.47	0.003
**Model 1b: Effect of craving and item drug relatedness on bids, controlling for study day (24** **h since/after methadone)**						
AIC	46292					
BIC	46371					
Num. of observations	9776					
		***B***^b^	***SE***	***t*–stat**	***df*** ^c^	***P*–value**
(Intercept)		2.75	0.69	3.99	28.91	0.0004
Study day (after methadone)		0.54	0.06	8.36	9746.80	<2.00 × 10^–16^
Drug relatedness		−1.94	0.47	−4.18	25.30	0.0003
Opioid craving (person-mean)		1.39	1.61	0.86	28.75	0.40
Opioid craving (person-centered)		1.57	0.21	7.50	9728.81	6.74 × 10^–14^
Drug relatedness × opioid craving (person-mean)		2.56	1.12	2.29	26.59	0.03
Drug relatedness × opioid craving (person-centered)		0.80	0.41	1.97	9720.89	0.049
**Model 2: Effect of craving, item drug relatedness, and item quantity on bids, controlling for study day (24** **h since/after methadone)**						
AIC	42075.3					
BIC	42219					
Num. of observations	9776					
		***B***^b^	***SE***	***t*–stat**	***df*** ^c^	***P*–value**
(Intercept)		0.98	0.43	2.28	30.39	0.03
Study day (after methadone)		0.54	0.05	10.39	9722.87	<2.00 × 10^–16^
Drug relatedness		−0.96	0.50	−1.93	36.25	0.06
Quantity		0.42	0.12	3.58	29.63	0.001
Opioid craving (person-mean)		0.36	1.01	0.36	29.96	0.72
Opioid craving (person-centered)		2.21	0.29	7.55	9687.18	4.72 × 10^–14^
Drug relatedness × quantity		−0.23	0.05	−4.97	9392.67	6.85 × 10^–7^
Drug relatedness × opioid craving (person-mean)		0.97	1.19	0.82	38.03	0.42
Drug relatedness × opioid craving (person-centered)		−0.30	0.62	−0.49	9681.15	0.63
Quantity × opioid craving (person-mean)		0.24	0.27	0.86	29.53	0.39
Quantity × opioid craving (person-centered)		−0.15	0.06	−2.69	9680.81	0.007
Drug relatedness × quantity × opioid craving (person-mean)		0.38	0.11	3.40	9349	0.0007
Drug relatedness × quantity × opioid craving (person-centered)		0.26	0.12	2.08	9680.81	0.04

^a^Results of linear mixed-effects regressions including random intercepts and random slopes for drug relatedness (both models) and quantity (Model 2) by participant and the listed predictors as fixed effects;

^b^Unstandardized coefficient. All ratings data are coded on a numeric 0–1 scale (possible values: 0–100% in 0.1% increments of the rating scale’s max value), quantity is coded on a numeric 1–8 scale (possible values: 1, 3, 5, and 8 units), bids are coded on a numeric 0–15 scale (possible values: $0–$15 in $0.02 increments), and study day is coded as a factor (24 h since or shortly after methadone);

^c^Degrees of freedom computed using Satterthwaite approximation.

## Results

### Opioid craving selectively increases drug-related valuation

**Fig. 3:**
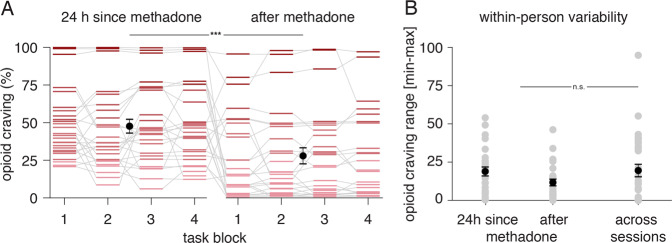
Opioid craving dynamics. **A** Momentary reported opioid craving (% of scale’s max) by study day (after 24 h from methadone dosing and shortly after dosing; randomized order) and task block (up to four) within a day. Craving was higher 24 h after methadone dosing and varied substantially both between- and within-person across the task blocks (intraclass correlation coefficient = 0.56). **B** Within-person variability in reported craving (range in opioid craving ratings; gray points show single-participant data) was comparable across blocks within a day to variability across days. ***P = 0.001, n.s. nonsignificant.

**Fig. 4:**
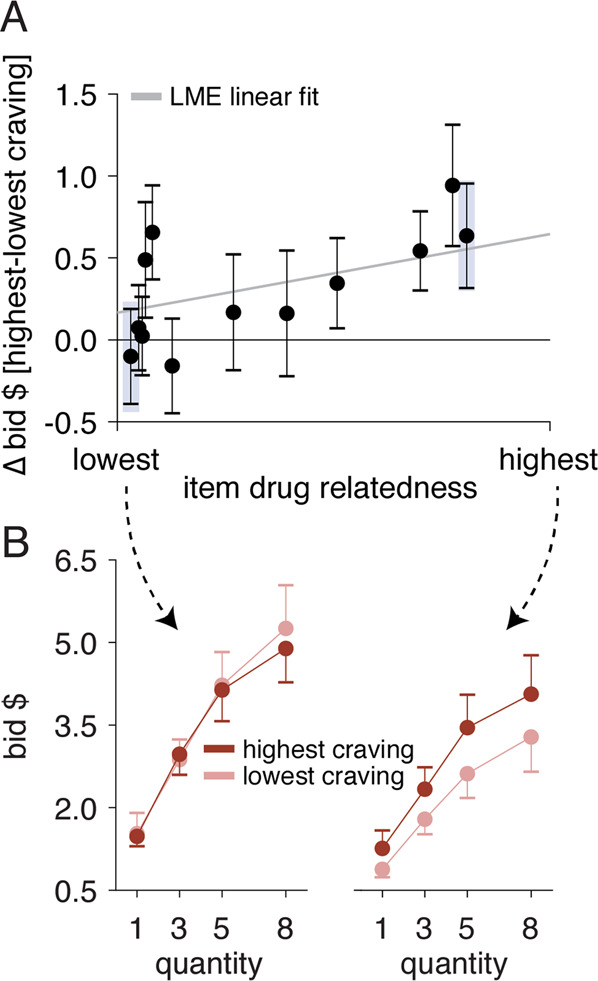
Observed pattern of craving effects in valuation. **A** Change in the subjective value (bid) for each of the task items on offer with opioid craving, ranked by the individually rated drug relatedness of each item across participants. **B** Subjective value (bids) for each quantity of the item with the lowest and highest drug relatedness in the choice set for each participant (shaded areas in **A**), at the lowest (peach), and highest (dark red) opioid craving task blocks for that participant. For visualization purposes, only data for each participant’s highest vs. lowest craving blocks (both panels) and highest vs. lowest drug-related items (**B**) are shown. The analyses reported in the text and tables used the full dataset. See Table 1 and Supplementary Information, Tables S3–S5.

Opioid craving was higher 24 h since vs. shortly after methadone dosing (*B* = 0.20, *SE* = 0.05, *t*_27.09_ = 3.67, *P* = 0.001; Fig. 3A) and varied substantially person-to-person as well as within-person, block-to-block (intraclass correlation coefficient = 0.56; Fig. 3B). To examine whether both between- and within-person craving effects may be present in participants’ concomitant valuation behavior, we first tested whether trial-by-trial bids varied with an item’s drug relatedness and the interaction of drug relatedness and craving (*Model 1a*). As expected, given participants were treatment-motivated, bids scaled negatively with drug relatedness: participants bid less for items they rated as more drug-related (*B* = −1.95, *P* = 0.0003; Table 1). However, we observed significant positive interactions between drug relatedness and both between- and within-person craving: participants who reported overall more craving bid more for items they rated as more drug-related (*B* = 2.56, *P* = 0.03), and moments of higher craving within-person also led to higher drug-related valuation (*B* = 1.21, *P* = 0.003; i.e., the negative relationship between bids and drug relatedness was lessened in moments of higher craving, Fig. 4A). These interaction effects were robust to study day (Table 1, *Model 1b*); they were also qualitatively similar for methadone and heroin craving when considered as separate forms of craving but were not better captured by either alone (Table S3 and Supplementary Information).

Interestingly, while participants assigned higher value to the items they rated as more (generally) desirable (*B* = 2.73, *P* = 0.03), opioid craving as assessed between- or within-person did not interact with item desirability to increase bids (*B* < | 3.90 |, *P* > 0.18) and controlling for item desirability did not modify the relationship between craving and drug relatedness (*P* < 0.05; Table S4). That is, craving appears to interact specifically with drug-related valuation and not general valuation.

These effects also appear to be specific to craving and are not explained by global changes in negative mood. While as expected, participants’ momentary negative mood correlated with their reported opioid craving (*B* = 0.30, *SE* = 0.08, *t*_175.15_ = 3.72, *P* = 0.0003), negative mood as assessed between- or within-person did not interact with item drug relatedness to increase bids (*B* < | 0.88 |, *P* > 0.59) and controlling for negative mood did not modify the relationship between craving and drug relatedness (*P* < 0.03; Table S4).

### Opioid craving increases drug-related valuation through a multiplicative gain-like mechanism

Our earlier work [22] indicated craving operates via a multiplicative-gain process, increasing subjective values placed on a class of goods based on the quantity of those goods (Fig. 1). If that is true for drug craving, then craving would increase not just the value of a single item but would “tilt” the entire curve that relates increasing quantities of drug-related items to subjective value. While somewhat counter-intuitive, this is a specific prediction of our earlier work, and it allows us to make strong mechanistic statements about value representation and craving.

To test this prediction, we included item quantity and its interaction with drug relatedness and craving as additional predictors of participants’ trial-to-trial bids (*Model 2*, Table 1). We found significant 3-way interactions that were again present at both the between- and within-person levels (*B* > 0.26, *P* < 0.04): participants who reported overall more craving bid disproportionately more for items rated as more drug-related specifically when these items were offered in higher quantities, and moments of higher craving also led to higher drug-related valuation for higher quantity offers (Fig. 4B). This suggests craving is reflected in drug-related valuation regardless of quantity but that this effect is stronger with increasing unit size, consistent with a pattern of escalating seeking behavior during craving.

In exploratory analyses, to examine more subtle changes in the relationship between quantity and bids, we fit a power function to participants’ data, for each item at each session and block, which was defined by a linear slope term (ω) and curvature (α). This permitted testing two possible ways that craving might scale drug-related valuation: multiplicatively, as evidenced by a shift in the ω term, or exponentially, as evidenced by a shift in the α term. Assessing these possibilities is important because an exponential shift would indicate craving fundamentally changes the valuation process (akin to a change in the internal value or utility function), while a multiplicative shift would indicate this process is preserved but scaled (akin to a gain-like effect on value). Consistent with a multiplicative shift, we observed a positive interaction between drug relatedness and within-person opioid craving on ω (*B* = 0.59, *P* = 0.048): ω was higher for items rated as more drug-related and when participants reported experiencing higher craving. We found nonsignificant between-person effects of craving for ω, and nonsignificant between- and within-person effects for the curvature parameter (α) suggesting that the core mechanism for placing values on goods remains unchanged during craving (Table S5).

## Discussion

We examined whether opioid craving, like food craving in health, operates in a selective manner in valuation, which may help to clarify how craving works to bias decisions toward drug-seeking actions and away from other valuable, and healthier, alternatives in treatment-engaged people with OUD. Applying a neuroeconomic framework that captured participants’ in-the-moment valuation of choice options spanning a continuous “drug relatedness” dimension, we found that opioid craving increased the relative subjective value of those options judged as more drug-related, and disproportionately so when they were offered in higher quantities. These effects were observed at both the between- and within-person levels, indicating higher “cravers” had higher drug-related valuation, and that, critically, moments of higher craving within-person also led to higher drug-related valuation. While fluctuation in negative mood state was, as predicted, associated with craving, negative mood did not have a comparable, direct effect in drug-related valuation. Collectively, these data suggest a shared (and outcome-specific) normative valuation process underlies drug and food craving, and provide specific predictions about the mechanism by which craving might interact with subjective valuation behaviorally and neurally.

The concept of “relative value” is central to theories of addiction grounded in behavioral economics [52, 53] and has been the focus of much work in other pertinent domains in addiction such as diagnosis and prognosis [54–65]. However, the specific effect of craving in drug- vs. nondrug-related valuation has received comparatively less attention. Using a design that specifically permitted assessing how craving operates along a drug relatedness dimension, we observed that craving positively interacted with this dimension, scaling subjective value for options that were deemed more (personally) drug-related. Further, we observed this effect regardless of an option’s general desirability, which was confirmed by (1) orthogonalizing drug relatedness and desirability in our choice set construction, and by (2) directly assessing value changes as a function of both drug relatedness and desirability. These data suggest craving acts on drug-related associative mechanisms, rather than through a general reinforcement process, and can help explain how craving specifically drives choice behavior toward the object of craving (and cues associated with drug-seeking and use).

Notably, craving had a multiplicative–gain-like–effect, amplifying participants’ assigned value for more drug-related options when these options were available in higher quantities. This can explain escalating drug-seeking behavior seen clinically with craving [4, 7, 19, 20] and in animal models of related constructs [66, 67]. It also provides a specific algorithmic process for future neurobiological work on craving which can be well tied to our basic understanding of the brain’s valuation circuitry. Neural circuits for craving and cue reactivity overlap with those generally involved in value-based decision-making, in particular the ventromedial prefrontal cortex and ventral striatum [68, 69], which receive dense dopaminergic inputs widely implicated in addiction [67], subjective craving responses [70, 71], and neural gain control [72, 73]. Interestingly, the topographical distribution of value codes within this circuitry, and especially ventral prefrontal cortex, appears to be at least partly outcome- (or attribute-category) specific [74–77]. We can speculate that the behavioral selectivity of craving in drug-related valuation (or similarly rated snacks foods in the case of food craving, as described previously) could stem from (possibly dopamine-dependent) modulation of outcome-specific value representations within this circuitry or at inputs to it, allowing for selective *hyper-*valuation with craving, as previously found with selective *de-*valuation [78].

While opioid craving in the current study and food craving in our previous work seem to operate in valuation via a shared mechanism, we also observed some notable differences. In our food study, healthy participants were willing to pay more for craved foods they found generally desirable; in contrast, treatment-engaged patients with OUD showed craving-modulated valuation behavior that was exclusively tied to the drug relatedness of the options on offer, irrespective of their general desirability. Thus, craving in addiction may transiently decouple desirability from valuation, in line with prominent habit-based theories of addiction and drug choice [79, 80] (although see [81, 82]). Further, although drug relatedness was negatively associated with subjective value in our treatment-engaged sample, craving seemed to flatten this beneficial response, highlighting the potential for risk for reuse and relapse during craving even under effective treatment.

Also distinct from food craving, which can be positively motivating [83], opioid craving is typically experienced as a negative affective state [36–41]. To determine the influence of concomitant negative mood, we continuously assessed participants’ current stress, happiness, and boredom. Although negative mood correlated positively with opioid craving, mood state shifts did not explain the relationship between craving and drug-related valuation, echoing prior work showing incidental and diffusive effects of mood in decision-making [42, 43], unlike the specific effect we observed with craving, which narrowed and focused motivation specifically toward the object of craving.

Several important limitations should be considered for future research. First, we relied on a single-item measure of craving as a proxy of craving more generally. Such single-item measures, while commonly used to evaluate in-the-moment experience, may fail to capture the multifaceted nature of craving, particularly in treatment-seekers where perceived ability to *control* want or desire may feature prominently [19, 84]. It would be prudent for future research to capture these additional facets in drug-related valuation, as well as determine how craving-modulated valuation changes over the treatment cycle from active use through treatment stabilization and in relation to actual drug-use behavior. Second, our sample was primarily male, reflecting known biases in treatment utilization and availability for women [85]. Given research suggesting sex-specific susceptibility to opioid craving and reuse [86], this sampling bias may contribute to an overall underestimation of the reported effects in women.

In summary, our results point to a signature of craving that can be applied to addiction specifically but used to describe craving more generally—one that is selective, operates on dimensional attribute-based similarity, and that is multiplicative of subjective value. Selectivity is indicated by the increased valuation of options closely associated with the object of craving (i.e., drugs or food), dimensionality by the way these options are valued based on their features (i.e., proximity to what is being craved), and multiplication by the scaling effect of quantity. From a clinical viewpoint, our experimental approach may be useful to uncover the cognitive processes underlying persistent craving in patients receiving gold-standard OUD treatments. Given the recent push for craving to be more clearly defined, measured, and incorporated into treatment targets for OUD [19, 84], our experimental approach presents a promising step in the development of measures that can both track craving over time and assess the effectiveness of interventions that aim to selectively block its effect on drug-related valuation.

## Supplementary information

Supplemental Material
