# Supplementary material for: A neuroeconomic signature of opioid craving: How fluctuations in craving bias drug-related and nondrug-related value
Source: Neuropsychopharmacology. 2021 Dec 16;47(8):1440–8. doi: 10.1038/s41386-021-01248-3 (PMC9205977; doi:10.1038/s41386-021-01248-3)
Supplement: Supplementary file 1 — Supplemental Material [file 41386_2021_1248_MOESM1_ESM.pdf]

SUPPLEMENTARY INFORMATION

A neuroeconomic signature of opioid craving: how fluctuations in craving bias drug-related and nondrug-related value

Kathryn Biernacki[1], Silvia Lopez-Guzman[2,3], John C. Messinger[2,4], Nidhi V. Banavar[2,5], John Rotrosen[6], Paul W. Glimcher[2,6*], Anna B. Konova[1,2*†]

[1]Department of Psychiatry, University Behavioral Health Care, and the Brain Health Institute, Rutgers University–New Brunswick, Piscataway, NJ 08855
[2]Neuroscience Institute, NYU Grossman School of Medicine, New York, NY 10016
[3]Grupo de Investigación en Neurociencias (NeURos), Escuela de Medicina y Ciencias de la Salud, Universidad del Rosario, Bogotá, Colombia, 111221
[4]Harvard Medical School, Boston, MA, 02115
[5]Department of Cognitive Sciences, University of California–Irvine, Irvine, CA 92697
[6]Department of Psychiatry, NYU Grossman School of Medicine, New York, NY 10016
*These authors contributed equally.

[†]**Correspondence**: Anna B. Konova PhD, Department of Psychiatry, University Behavioral Health Care, and the Brain Health Institute, Rutgers University–New Brunswick, 671 Hoes Lane West, Room D437, Piscataway, NJ 08855; tel.: 732-867-9720; e-mail: anna.konova@rutgers.edu

Supplementary Text
Figure S1
Tables S1-S5

Supplementary Text

Pre-task ratings (Day 1)

On *Day 1*, participants completed screening procedures and a series of ratings tasks that were later used to construct personalized choice sets for each individual. No specific instructions were given to participants about when to complete this session relative to their methadone dosing. Nevertheless, 27 out of 29 participants completed the session shortly after having received their daily methadone dose, while 2 participants completed it ~24 hours since the last dose. These participants' *Day 1* ratings data did not differ from the group's for the final selected choice sets for the task completed on *Days 2* and *3* (drug relatedness: B=–0.14, SE=0.14, $t_{28.99}$=–1.01, P=0.32; general desirability: B=–0.20, SE=0.16, $t_{28.99}$=–1.26, P=0.22, see below).

At this session, participants rated each of 40 consumer items and snack foods (see **Fig. 2A** in the main text) on multiple dimensions, including: drug relatedness, general desirability, frequency of consumption/familiarity, and tastiness and healthiness (of snack foods only). These ratings were completed twice for the drug relatedness dimension (which were then averaged for each item). All other ratings were completed once for each of the other dimensions. All ratings scales were unit-less and used a continuous slider bar that recorded the cursor position in high-resolution (0.1% increments relative to the total bar length). All ratings data were transformed to 0–1 (reflecting the final position of the cursor relative to the max possible position). The specific instructions given to participants are detailed below.

Drug relatedness: *In this part, we are going to show you all the different items, one at a time. We ask that you think about how much each item reminds you of heroin/pills, thinking about getting high, being high, or recovering from using drugs. That is, if you came across this item, how likely are you to think of using heroin and other drugs, from "Not at all likely" to "Extremely likely"?*

General desirability: *In this part, we are going to show you all the different items, one at a time. We ask that you think about the DESIRABILITY of each item to you in general, from "Not at all desirable" to "Extremely desirable".*

Frequency of consumption/use: *In this part, we are going to show you all the different items, one at a time. We ask that you think about HOW OFTEN you use or consume each item, from "Not often at all" to "All the time". For example, if you consume or use a given item once every 5 years, your rating should be close to "Not often at all". If it is every day, your rating should be close to "All the time".*

Tastiness (snack foods only): *In this part, we are going to show you all of the snacks, one at a time. We ask that you think about the TASTINESS of each snack to you, without regard for its healthiness, from "Not at all tasty" to "Extremely tasty".*

Healthiness (snack foods only): *In this part, we are going to show you all of the snacks, one at a time. We ask that you think about the HEALTHINESS of each snack, without regard for its tastiness, from "Not at all healthy" to "Extremely healthy".*

Choice set algorithm (Day 1)

The subset of 12 consumer items/snack foods used in the task completed on *Days 2* and *3* was individualized for each participant from a broader set of 40 items that were identical for all (see main text **Fig. 2A** and **Fig. S1**). These 40 items were selected by the investigators, in part informed by an informal pre-study survey conducted in the same methadone treatment program that inquired about everyday items that patients associated with opioid consumption and that they would pay any amount of money to purchase (even pennies). From this informal survey, we obtained items that had an evident linkage to opioid use such as hypodermic needle syringes. We also obtained consistent reports of items that may not be usually found in the list of stimuli of traditional cue reactivity studies, but that participants associated with their opioid consumption (e.g., 8 oz bottles of Poland Spring water). Such items may not traditionally be considered drug-related (compared to others like syringes and lighters) and were as such included in the list of 40.

The individualization of the choice subset of 12 items was achieved by means of an algorithm that randomly and iteratively selected 12 items (6 snack food and 6 non-food items) from the broader list of 40 and computed the correlation between their drug-relatedness and their desirability as rated by that participant. The algorithm identified the set of 12 items with maximal standard deviation for drug relatedness and then computed the partial correlation between drug relatedness and desirability across that set controlling for item type (food vs. non-food). The algorithm also ran a paired *t*-test for both ratings by item type. Unless the significance level of the partial correlation and the *t*-test both exceeded 0.25 (critical $P>0.25$), equivalent to a critical correlation coefficient $R<|0.2058|$, the algorithm would continue through the remaining combinations of 12-item sets removing the current set from consideration. The fixed effect correlation between drug relatedness and general desirability for the final 12-item sets across all participants ($P=0.267$) confirmed orthogonalization procedure was successful.

In-task ratings (Days 2 and 3)

On *Days 2* and *3*, participants completed the willingness-to-pay task. The task included three trial types: bid trials, desire rating trials, and current mood/craving rating trials. In *bid trials*, participants were asked to indicate their willingness-to-pay in the current moment for each item in their 12-item choice set, offered one at a time in one of four quantities. In *desire rating trials*, participants were asked to indicate their desire in the current moment for each item, again offered in different quantities. In *mood rating trials*, participants were asked to report on their current feelings of boredom, stress, and happiness. In *craving rating trials*, current desire for heroin and methadone were used to evaluate overall opioid craving. The different mood and craving rating trials were identified by a picture cue corresponding to the rating type. All ratings scales were unit-less and used a continuous slider bar that recorded the cursor position in high-resolution (0.1% increments relative to the total bar length). All ratings data were transformed to 0–1 (reflecting the final position of the cursor relative to the max possible position). The specific instructions given to participants for these ratings are detailed below.

Bid trials: *In these trials, you will see an item and indicate how much you would be willing to pay to have the item(s) RIGHT NOW. You might see the same items(s) more than once. However, we are only interested in your bid for the items(s) in the current moment. Do not to worry about your previous bids for the same item(s) or plan for what you might do next time. […] Your bid should indicate what is the maximum amount of $ out of your $15 you would pay RIGHT NOW for the item(s) shown for the real chance of actually getting the item(s) RIGHT NOW. […] On days 2 and 3 you will get bonus rewards. These rewards will be determined by your responses on bid trials. You are going to place bids for many items, and possibly more than once for each. You should always place yours bids based on how you feel in the current moment. This because the task can end early. It can last anywhere from a few minutes to over an hour. When the task ends, we are going to pick only ONE of your recent bids. We will then compare your bid for the item(s) shown on that one decision with a randomly chosen "selling price". If your bid is higher, you will get to buy the item(s) at the lower selling price and keep the change. Your bonus will then be the item(s) plus the left over $. If your bid is lower, you won't get to buy the item(s). Your bonus will then be no item(s) plus $15.*

Desire rating trials: *In these trials, you will see an item and rate how much would like to have this item RIGHT NOW. You might see each item more than once. However, we are only interested in your rating for each item in the current moment. Do not to worry about your previous ratings for the same item or plan for what you might do next time. […] Your desire rating should indicate how much you want the item(s) shown RIGHT NOW, without any*

consequences for whether or not you can actually have the item(s) RIGHT NOW. [...] These desire ratings have no impact on your bonus rewards so please answer honestly. They are however a crucial part of the experiment so please also be as accurate as possible.

Mood trials: *How STRESSED are you right now? How HAPPY are you right now? How BORED are you right now?*

Craving trials: *How much do you want HEROIN right now? How much do you want METHADONE right now?*

In the main data analyses, heroin and methadone craving ratings were averaged to obtain a single assessment of opioid craving. Given that each task block (lasting on average for 12-15 minutes) had only a single randomly interspersed craving rating per drug type and a single mood/affective state rating per mood/affective state type (see main text, **Fig. 2B**), grouping these into two composite measures provided a less noisy estimate of the aggregate level state of the person across the duration of the entire block. When we re-analyze the data separating the heroin and methadone craving ratings, we find (1) qualitatively similar results with heroin and methadone craving as separate measures, but (2) comparably worse overall model performance. As shown in **Table S3**, like the composite opioid craving measure (see **Table 1**), both heroin and methadone craving interact significantly with drug relatedness to influence valuation. This is observed when the two are used as predictors in separate models or in the same model, and when additionally controlling for study day (shortly after or 24 hours since last methadone dose). However, the relative contribution of between-person versus within-person effects differs somewhat between the two (with within-person effects having somewhat larger effect for heroin craving). Importantly, these models, which break out craving into heroin and methadone craving, do not provide a better fit to the data over the composite opioid craving model [and if anything, they provide worse fits, especially when accounting for study day: ΔBIC of –14.7 to –36.1 in favor of the composite model]. For parsimony, we therefore focused our main analyses on the composite opioid craving measure.

Bid realization for bonus payment (Days 2 and 3)

At the conclusion of the willingness-to-pay task at each task session (*Days 2* and *3*), a single recent (from the last completed block) bid trial was selected for realization. To determine a participant's bonus, we implemented a standard Becker-DeGroot-Marschak (BDM) auction whereby the selected bid b was compared against a randomly drawn selling price p. To determine p, chips numbered from \$0 to \$15 in \$0.02 increments were drawn from a bag. If $b \geq p$

(the price on the chips), the item(s) offered on the selected trial could be purchased for the price *p*. If $b < p$, however, the item(s) could not be purchased. Thus, in the first case the bonus consisted of the item(s) offered plus any unspent money, and in the second case, no item(s) plus the full endowment. The BDM procedure is widely used in laboratory economic studies because it elicits a participant's "true" subjective value for each item, and in our case for a given moment in time. Its design ensures the best strategy for participants is to report their maximum willingness to-pay price, without over- or under-estimating this amount.

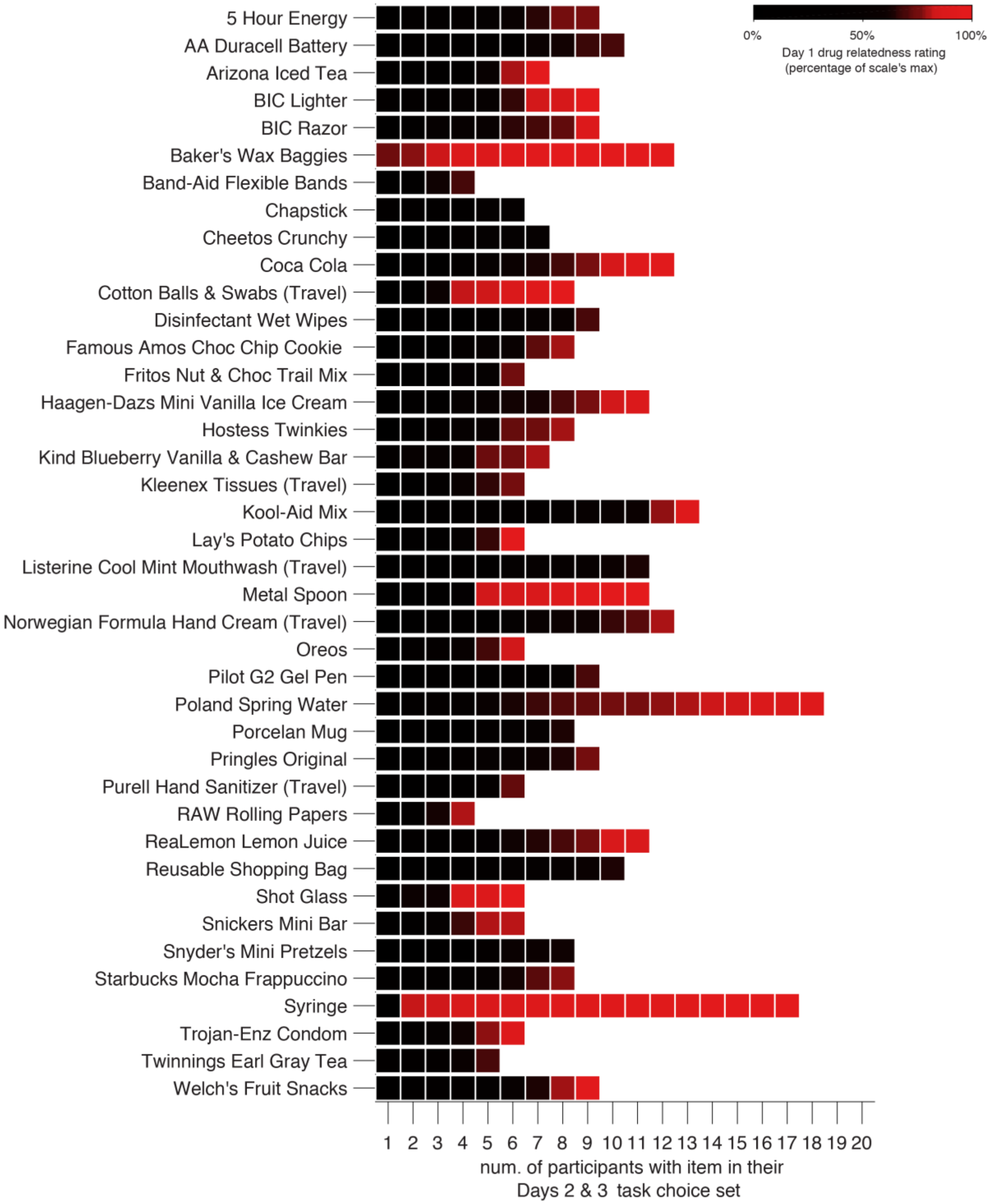

Fig. S1. Items comprising individual choice sets sorted by subjective drug relatedness. On *Day 1*, participants rated each of 40 common consumer items and snack foods on their subjective drug relatedness and general desirability. These ratings were used to sub-select a 12-item choice set for each participant for the task completed on *Days 2* and *3* (see main text and **Fig. 2A**). Shown here are the number of participants who had each item from the initial 40-item fixed set in their personalized 12-item sub-set (note that one participant inadvertently completed

the task with 11 instead of 12 items, leading to 347 unique participant-item combinations: 28×12 + 1×11). Each item was represented in at least one participant's choice set. However, this could be for different reasons: the same item could belong to a choice set because it was rated as low *or* high on drug relatedness by a given participant. While for a few items there was consensus that the item was "especially" drug-related (e.g., rated as being >75% of the scale's max in drug relatedness by most participants), for most others there was high degree of idiosyncrasy in these subjective judgements.

Table S1. Demographic and clinical characteristics of the study sample

**Demographics**	
Total *N*	29
Age (years)	47.38 (*SD*=11.53)
Sex (male/female)	26/3
Race (African American/Caucasian)	9/20
Ethnicity (Hispanic/Non-Hispanic)	8/21
Education (years)	12.66 (*SD*=1.61)
Income (monthly)	$0–$2100 (Median=$690)
Non-verbal IQ [*]	92.44 (*SD*=12.85)
**Psychiatric & Substance Use History**	
DSM5 Opioid Use Disorder total symptom count	7.24 (*SD*=3.07)
Heroin use (lifetime years) [†]	14.11 (*SD*=11.61)
Route of administration	
Nasal	31%
Intravenous	28%
Mixed	41%
Length of current treatment (days)	6.57–2626.52 (Median=83)
Previous drug treatment episodes [‡]	7.69 (*SD*=8.48)
Methadone dose (mg)	92.24 (*SD*=31.33)
Secondary Substance Use Disorders	
Cocaine	52%
Benzodiazepine	21%
Other	41%
Nicotine smoker	86%
Nicotine dependence (FTND)	3.52 (*SD*=1.83)
Depression (BDI-II) [§]	16.07 (*SD*=12.73)
Anxiety (BAI) [‖]	17.62 (*SD*=12.04)

[*] Standardized score from the Kaufman Brief Intelligence Test (K-BIT). Normative data suggest scores between 85–115 constitute the average nonverbal IQ of the population;
[†] Lifetime use from the Addiction Severity Index (ASI);
[‡] Previous inpatient (detox, residential) or outpatient treatment based on the ASI;
[§] Beck Depression Inventory (BDI-II). Depression severity cut-offs for the BDI-II are as follows: 0–13 minimal, 14–19 mild, 20–28 moderate, and 29–63 severe;
[‖] Beck Anxiety Inventory (BAI). Anxiety severity cut-offs for the BAI are as follows: 0–9 minimal, 10–16 mild, 17–29 moderate, and 30–63 severe.

Table S2. **Baseline measures collected at the beginning of each study day/session**

	**Day 1 – screening & choice set selection session**	**Day 2 or 3 – 24 h since methadone dosing**	**Day 2 or 3 – shortly after methadone dosing**
Heroin Craving Questionnaire-Short Form 14 (HCQ-Now) (1–7)	2.46 (*SD*=0.79)	2.34 (*SD*=0.91)	2.29 (*SD*=0.83)
Subjective Opioid Withdrawal Scale (SOWS) (0–64) *	7.70 (*SD*=7.30)	7.77 (*SD*=8.11)	5.58 (*SD*=5.31)
State-Trait Anxiety Inventory (STAI-State) (20–80)	43.10 (*SD*=14.66)	41.77 (*SD*=13.56)	42.00 (*SD*=15.68)
Recent opioid use – past 7 days (% endorse)	62.07%	50.00%	50.00%
Hours of sleep	5.19 (*SD*=2.31)	5.16 (*SD*=3.10)	5.78 (*SD*=2.34)
Current hunger (1–10)	4.14 (*SD*=2.47)	4.77 (*SD*=2.47)	3.92 (*SD*=2.17)
Current thirst (1–10)	4.24 (*SD*=2.05)	4.65 (*SD*=2.38)	4.65 (*SD*=2.18)
In-task reported opioid craving (composite measure, 0–1) †	–	0.48 (*SD*=0.24)	0.28 (*SD*=0.28)

* Subjective Opioid Withdrawal Scale (SOWS). Withdrawal severity cut-offs for the SOWS are as follows: 1–10 mild, 11–20 moderate, and 21–30 severe. Withdrawal differs significantly between the two task days (24 h from the last dose of methadone vs. after methadone dosing, P=0.046);

† In-task reported craving averaged across blocks comprising each study day and across heroin and methadone craving (response to single-item probe, "How much do you want [heroin/methadone] right now?"). In-task craving differs significantly between the two task days (24 hours from the last dose of methadone vs. after methadone dosing, P=0.001; see main text and **Fig. 3**). In-task craving ratings correlate with the 14-item HCQ-Now multidimensional baseline assessment of craving ($P=1.1\times10^{-14}$), suggesting this single-item measure well-approximates broader definitions of craving.

Table S3. Association of patients' level of current reported heroin and methadone craving and item drug-relatedness, with bids *

Model: Effect of heroin craving and item drug relatedness on bids, controlling for study day (24 h since/after methadone)						
AIC	46306.6					
BIC	46385.7					
Num. of observations	9776	***B***†	***SE***	***t*–stat**	***df*** ‡	***P*–value**
(Intercept)		3.12	0.52	5.96	28.95	1.08×10^{-6}
Study day (after methadone)		0.35	0.06	6.15	9742.09	8.33×10^{-10}
Drug relatedness		–1.36	0.37	–3.69	23.69	0.001
Heroin craving (person-mean)		0.81	1.35	0.60	28.79	0.55
Heroin craving (person-centered)		1.28	0.21	6.06	9721.10	1.39×10^{-9}
Drug relatedness × heroin craving (person-mean)		1.27	1.00	1.27	26.39	0.21
Drug relatedness × heroin craving (person-centered)		1.33	0.46	2.92	9719.55	0.004
**Model: Effect of methadone craving and item drug relatedness on bids, controlling for study day (24 h since/after methadone)**						
AIC	46328					
BIC	46407.1					
Num. of observations	9776	***B***†	***SE***	***t*–stat**	***df*** ‡	***P*–value**
(Intercept)		2.68	0.74	3.63	28.98	0.001
Study day (after methadone)		0.54	0.07	7.83	9748.29	5.57×10^{-15}
Drug relatedness		–2.11	0.49	–4.34	26.70	0.0002
Methadone craving (person-mean)		1.29	1.45	0.89	28.76	0.38
Methadone craving (person-centered)		1.02	0.16	6.47	9732.14	1.02×10^{-10}
Drug relatedness × methadone craving (person-mean)		2.39	0.95	2.52	26.78	0.02
Drug relatedness × methadone craving (person-centered)		0.48	0.30	1.60	9721.23	0.11
**Model: Effect of heroin craving, methadone craving, and item drug relatedness on bids, controlling for study day (24 h since/after methadone)**						
AIC	46291.3					
BIC	46399.1					
Num. of observations	9776	***B***†	***SE***	***t*–stat**	***df*** ‡	***P*–value**
(Intercept)		2.68	0.74	3.62	28.93	0.001
Study day (after methadone)		0.51	0.07	7.42	9747.87	1.25×10^{-13}
Drug relatedness		–2.14	0.49	–4.38	26.45	0.0002
Heroin craving (person-mean)		0.31	1.53	0.21	28.82	0.84
Heroin craving (person-centered)		0.88	0.24	3.71	9720.79	0.0002
Methadone craving (person-mean)		1.11	1.65	0.68	28.74	0.51
Methadone craving (person-centered)		0.68	0.18	3.85	9730.61	0.0001
Drug relatedness × heroin craving (person-mean)		0.33	1.00	0.34	27.02	0.74
Drug relatedness × heroin craving (person-centered)		1.37	0.55	2.47	9720.67	0.01
Drug relatedness × methadone craving (person-mean)		2.25	1.04	2.16	26.39	0.04
Drug relatedness × methadone craving (person-centered)		–0.18	0.36	–0.50	9721.04	0.62

[*] Results of linear mixed-effects regressions including random intercepts and random slopes for drug relatedness by participant and the listed predictors as fixed effects;
[†] Unstandardized coefficient. All ratings data are coded on a numeric 0–1 scale (possible values: 0–100% in 0.1% increments of the rating scale's max value), bids are coded on a numeric 0–15 scale (possible values: $0–$15 in $0.02 increments), and study day is coded as a factor (24 hours since or shortly after methadone);
[‡] Degrees of freedom computed using Satterthwaite approximation.

Table S4. Association of patient's level of current reported opioid craving, negative mood, and item drug relatedness and desirability with bids *

**Model: Effect of craving, item drug relatedness, and item desirability on bids, controlling for study day (24 h since/after methadone)**						
AIC	45526.5					
BIC	45648.7					
Num. of observations	9776	***B***†	***SE***	***t*–stat**	***df*** ‡	***P*–value**
(Intercept)		2.09	0.76	2.74	28.39	0.01
Study day (after methadone)		0.54	0.06	8.74	9706.47	$<2.00\times10^{-16}$
Drug relatedness		–1.73	0.51	–3.38	20.52	0.003
General desirability		2.73	1.22	2.24	27.96	0.03
Opioid craving (person-mean)		2.33	1.80	1.30	28.37	0.21
Opioid craving (person-centered)		1.55	0.25	6.11	9689.32	1.01×10^{-9}
General desirability × opioid craving (person-mean)		–3.90	2.84	–1.37	28.07	0.18
General desirability × opioid craving (person-centered)		0.04	0.36	0.11	9684.58	0.91
Drug relatedness × opioid craving (person-mean)		2.55	1.23	2.08	21.92	0.049
Drug relatedness × opioid craving (person-centered)		0.80	0.39	2.05	9684.89	0.04
**Model: Effect of craving, negative mood, and item drug relatedness on bids, controlling for study day (24 h since/after methadone)**						
AIC	46279.7					
BIC	46387.5					
Num. of observations	9776	***B***†	***SE***	***t*–stat**	***df*** ‡	***P*–value**
(Intercept)		2.86	1.19	2.41	28.83	0.02
Study day (after methadone)		0.48	0.07	7.15	9747	9.61×10^{-13}
Drug relatedness		–1.56	0.82	–1.89	24.87	0.07
Negative mood (person-mean)		–0.18	2.44	–0.07	28.87	0.94
Negative mood (person-centered)		0.77	0.21	3.72	9722	0.0002
Opioid craving (person-mean)		1.37	1.62	0.85	28.71	0.40
Opioid craving (person-centered)		1.24	0.22	5.56	9730	2.84×10^{-8}
Drug relatedness × negative mood (person-mean)		–0.88	1.59	–0.55	24.69	0.59
Drug relatedness × negative mood (person-centered)		–0.004	0.47	–0.009	9721	0.99
Drug relatedness × opioid craving (person-mean)		2.50	1.12	2.22	26.87	0.03
Drug relatedness × opioid craving (person-centered)		0.98	0.42	2.35	9721	0.02

* Results of linear mixed-effects regressions including random intercepts and random slopes for drug relatedness (both models) and general desirability (2nd model) by participant and the listed predictors as fixed effects;

† Unstandardized coefficient. All ratings data are coded on a numeric 0–1 scale (possible values: 0–100% in 0.1% increments of the rating scale's max value), bids are coded on a numeric 0–15 scale (possible values: $0–$15 in $0.02 increments), and study day is coded as a factor (24 hours since or shortly after methadone);

‡ Degrees of freedom computed using Satterthwaite approximation.

Table S5. Association of patients' level of current reported opioid craving and item drug-relatedness with the slope and curvature of the utility curve for each item offered *

**Model: Effect of craving and item drug relatedness on ω parameter (slope), controlling for study day (24 h since/after methadone)**						
AIC	6840.6					
BIC	6904.5					
Num. of observations	2444	***B***†	***SE***	***t*–stat**	***df*** ‡	***P*–value**
(Intercept)		1.26	0.32	3.88	29.26	0.0005
Study day (after methadone)		0.27	0.05	5.52	2413.66	3.70×10^{-8}
Drug relatedness		–0.85	0.26	–3.29	26.09	0.003
Opioid craving (person-mean)		0.50	0.76	0.66	28.82	0.51
Opioid craving (person-centered)		1.05	0.15	6.75	2398.77	1.91×10^{-11}
Drug relatedness × opioid craving (person-mean)		0.97	0.62	1.55	26.59	0.13
Drug relatedness × opioid craving (person-centered)		0.59	0.30	1.98	2392.68	0.048
**Model: Effect of craving and item drug relatedness on α parameter (curvature), controlling for study day (24 h since/after methadone)**						
AIC	3682.3					
BIC	3746.1					
Num. of observations	2444	***B***†	***SE***	***t*–stat**	***df*** ‡	***P*–value**
(Intercept)		0.54	0.07	7.66	32.67	8.80×10^{-9}
Study day (after methadone)		0.05	0.03	1.81	2425	0.07
Drug relatedness		–0.10	0.06	–1.61	181.9	0.11
Opioid craving (person-mean)		0.12	0.16	0.77	29.70	0.45
Opioid craving (person-centered)		–0.009	0.08	–0.01	2434	0.99
Drug relatedness × opioid craving (person-mean)		0.16	0.15	1.06	173	0.29
Drug relatedness × opioid craving (person-centered)		0.03	0.16	0.22	2416	0.83

* Results of linear mixed-effects regressions including random intercepts and random slopes for drug relatedness by participant and the listed predictors as fixed effects;
† Unstandardized coefficient. All ratings data are coded on a numeric 0–1 scale (possible values: 0–100% in 0.1% increments of the rating scale's max value), the fitted utility curve parameters are coded on a numeric 0–5 scale (continuous), and study day is coded as a factor (24 hours since or shortly after methadone);
‡ Degrees of freedom computed using Satterthwaite approximation.
